# Retinaler arteriovenöser Gefäßverschluss nach COVID-Impfung mit Vaxzevria® (AstraZeneca) – Eine Impfkomplikation oder nicht?

**DOI:** 10.1007/s00347-022-01598-3

**Published:** 2022-03-11

**Authors:** S. Groselli, K. Gabka, L. Bechstein, M. Ulbig

**Affiliations:** Klinik und Poliklinik für Augenheilkunde, Klinikum rechts der Isar, TU München, Ismaninger Str. 22, 81675 München, Deutschland

## Falldarstellung

### Anamnese

Ein 77-jähriger männlicher Patient stellte sich zur Zweitmeinung in unserer Klinik vor. Er berichtete von einer Visusminderung am linken Auge seit 1 Monat. Er gab an, dass die Beschwerden einen Tag nach seiner Corona-Impfung mit dem Impfstoff Vaxzevria® von AstraZeneca aufgetreten seien. Der Patient habe sich daraufhin direkt zu einem Augenarzt begeben. Dieser habe eine Fluoreszenzangiographie durchgeführt und einen retinalen zentralen Venenverschluss mit Makulaödem am linken Auge festgestellt. Daraufhin erhielt der Patient eine 2‑malige intravitreale Medikamenteneingabe (IVOM) mit Ranibizumab, worauf eine Besserung der Symptomatik auftrat. Eine dritte IVOM sei bereits geplant. Eine internistische Abklärung (Langzeitblutdruckmessung, Elektrokardiogramm, Doppler der hirnversorgenden A\rterien, Blutbild) war erfolgt und unauffällig.

### Klinischer Befund

**Figure 1:**
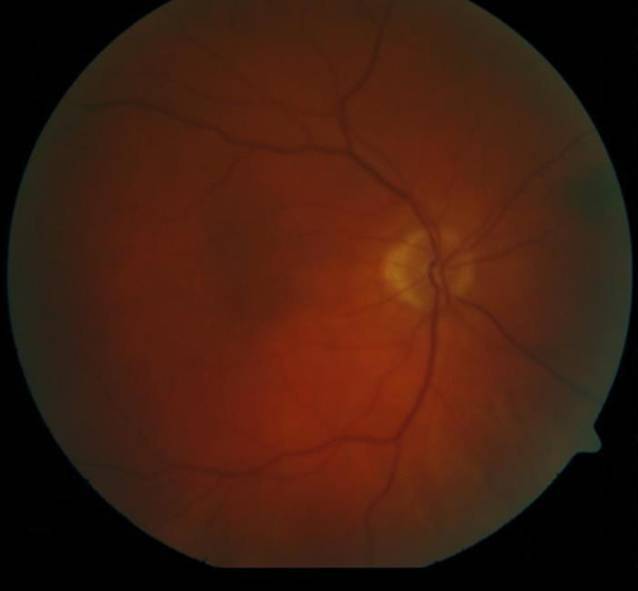

**Figure 2:**
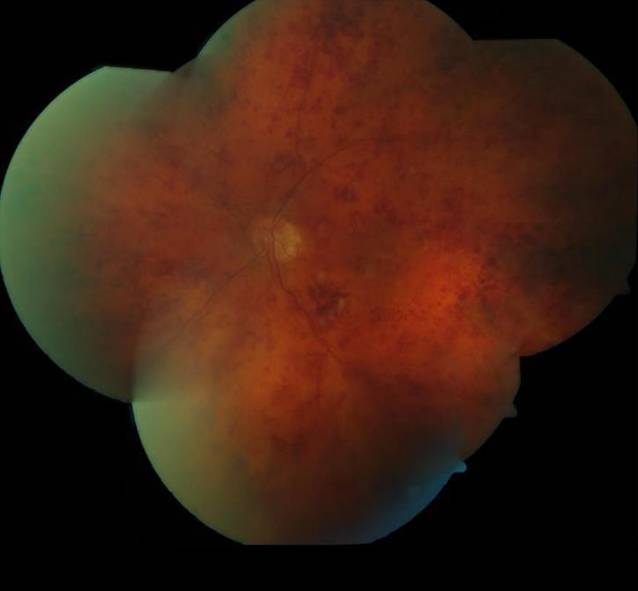

Unsere Untersuchungen zeigten bei einem bestkorrigierten Visus von 0,9 pp (+0,75 dpt sph. −0,75 dpt cyl. 114° A) am rechten Auge und 0,1 (+1,25 dpt sph. −0,50 dpt cyl. 166° A) am linken Auge und reizfreie vordere Augenabschnitte mit einer beidseits präprovecten Katarakt. Der Augeninnendruck war normoton. Fundoskopisch stellte sich am rechten Auge außer dezenten Kreuzungszeichen und einem älteren peripheren Netzhautforamen bei 1 Uhr mit angrenzenden vernarbten Lasernarben ein regelrechter Befund dar (Abb. 1). Am linken Auge war die Papille randscharf begrenzt und farbarm, die Gefäße waren tortuiert, Kreuzungszeichen, und es zeigten sich Punkt- und Fleckblutungen am gesamten Fundus. Die Netzhaut war allseits anliegend (Abb. 2).

### Diagnostik

**Figure 3:**
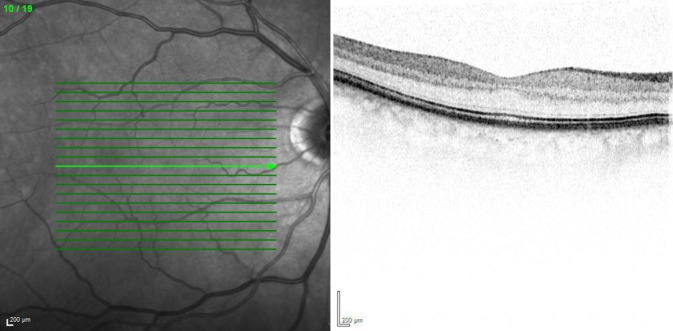

**Figure 4:**
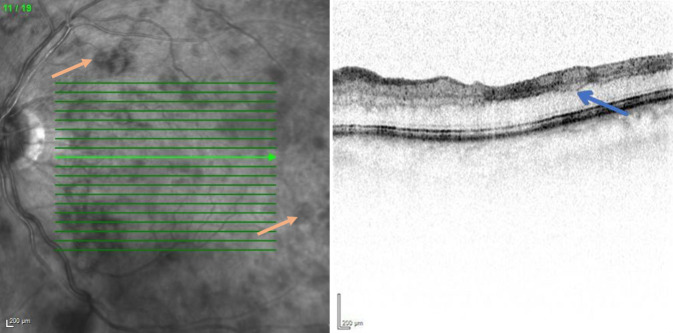

Wir führten eine Spectral-domain-optische Kohärenztomographie (SD-OCT) durch, in der wir am rechten Auge einen regelrechten Befund feststellen konnten (Abb. 3). Am linken betroffenen Auge zeigte sich eine ischämische Bande ohne intra- oder subretinale Flüssigkeit (Abb. 4).

**Figure 5:**
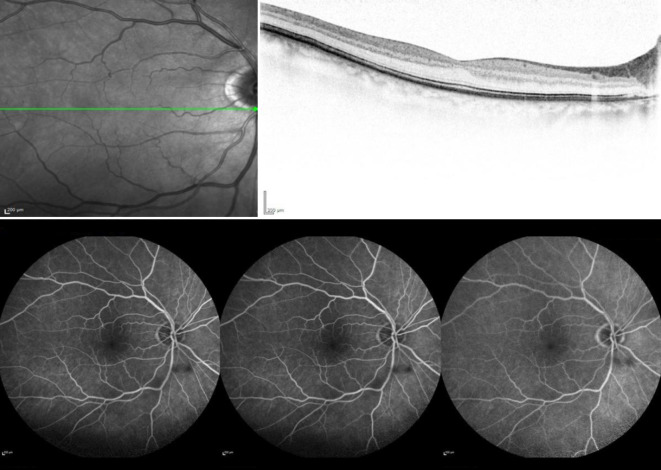

Des Weiteren führten wir eine Fluoreszenzangiographie (FLA) durch. Am rechten Auge zeigten sich ein regelrechter angiographischer Befund außer Hyperfluoreszenzen bei 1 Uhr peripher bei Zustand nach Laserkoagulation bei Netzhautforamen und ein Fensterdefekt. Insgesamt gab es keinen Hinweis auf eine Perfusionsstörung (Abb. 5).

**Figure 6:**
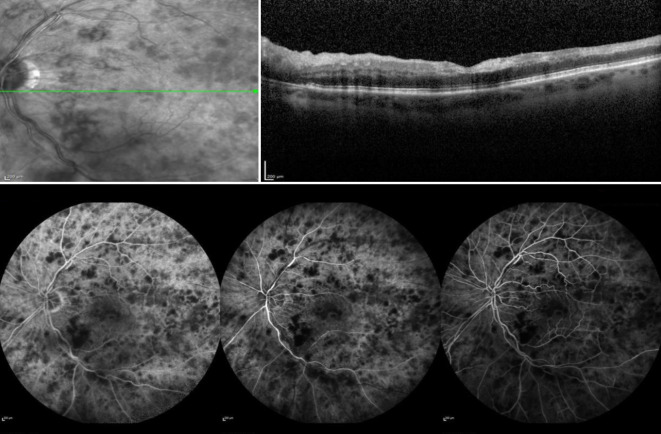

Am linken Auge stellten sich eine regelrechte Füllung der choroidalen Gefäße und flächenhafte fleckförmige Hypofluoreszenzen bei Blockadephänomen durch die intraretinalen Blutungen dar. Es zeigte sich eine zunehmende Füllung der arteriellen retinalen Gefäße. Im Bereich des oberen Gefäßbogens lag bei arteriellen Kaliberschwankungen der Verdacht auf eine Stenose vor. Peripher zeigten sich ischämische Areale und in der Spätphase ein regelrechtes Abklingen der Fluoreszenzphänomene (Abb. 6).

### Therapie und Verlauf

In Zusammenschau sämtlicher Befunde zeigte sich auf dem linken Auge ein Mischbild aus arteriovenösem Gefäßverschluss. Bei vorliegender Arbeitsdiagnose wurde eine Mild-scatter-pan-Laserkoagulation gezielt in den peripheren ischämischen Arealen empfohlen. Ein Thrombophiliescreening wurde abgenommen und war unauffällig.

Zusätzlich wurden weitere Kontrolluntersuchungen beim niedergelassenen Augenarzt empfohlen, bei dem sich unser Patient für die geplante dritte intravitreale Medikamenteneingabe (IVOM) vorstellen sollte.

## Diskussion

ChAdOx1 nCOV-19 ist ein rekombinanter Adenovirus-Vektorimpfstoff, der von der Universität Oxford entwickelt wurde. Er wird unter dem Handelsnamen Vaxzevria® vermarktet (früher AstraZeneca COVID-19-Impfstoff, AstraZeneca) [6].

Mehr als 200 Fälle thrombotischer Ereignisse unter insgesamt 34 Mio. mit ChAdOx1 nCoV-19 geimpften Personen wurden an die europäische Datenbank für Verdachtsfälle von unerwünschten Wirkungen (EudraVigilance) gemeldet. Nach der Untersuchung der gemeldeten Fälle stellte die European Medical Association (EMA) einen Zusammenhang zwischen ChAdOx1 nCoV-19 und ungewöhnlichen thrombotischen Ereignissen sowie begleitender Thrombozytopenie fest. Die ungewöhnliche klinische Konstellation von zerebraler venöser Sinusthrombose (CVST) und Thrombozytopenie wird inzwischen als impfstoffinduzierte immunthrombotische Thrombozytopenie (VITT) bezeichnet [1]. Obwohl die WHO und die EMA zu dem Schluss kamen, dass der Nutzen der Impfung mit ChAdOx1 nCoV-19 die mit Thrombose und Thrombozytopenie verbundenen Risiken überwiegt, wurden im März 2021 die Impfungen mit Vaxzevria® (AstraZeneca) vom deutschen Gesundheitsministerium aufgrund von Sicherheitsbedenken hinsichtlich eines erhöhten Thromboserisikos vorübergehend ausgesetzt. Nach der Bewertung der potenziellen Risiken und des Nutzens des Impfstoffs durch den Sicherheitsausschuss der EMA wurden die Impfungen ab Ende März 2021 wieder aufgenommen. Bis zu diesem Zeitpunkt hatte das Paul-Ehrlich-Institut (PEI) über 13 Fälle von Sinus- oder Hirnvenenthrombosen bei mehr als 1,6 Mio. verabreichten Impfdosen von Vaxzevria® (AstraZeneca) berichtet. Die Thrombosen waren 4 bis 16 Tage nach der Impfung bei 12 Frauen und 1 Mann im Alter von 20 bis 63 Jahren aufgetreten. Die Patienten wiesen zudem eine Thrombozytopenie auf, was ein immunologisches Ereignis als Ursache für die Thromboseneigung nahelegt. Ein wichtiger Pathomechanismus wurde inzwischen innerhalb der Gesellschaft für Thrombose- und Hämostaseforschung (GTH) aufgeklärt. Unter den verschiedenen möglichen Erklärungen für diese extrem seltenen thrombotischen Komplikationen wird eine autoimmunbedingte, heparininduzierte Thrombozytopenie diskutiert [2, 6]. Warum sich diese immunogene Thrombose jedoch bevorzugt in zerebralen Gefäßen manifestiert, ist derzeit noch unklar [5].

Obwohl die Inzidenz der Fälle anfänglich bei jungen Frauen höher war, sind in den neuesten Daten atypische Thrombosen gleichermaßen bei Männern und Patienten über 60 Jahren aufgetreten, obwohl die Inzidenz der Meldung bei jüngeren Patienten proportional höher bleibt [2].

Thrombotische Ereignisse treten nicht ausschließlich als intrakranielle Thrombosen auf, sondern können sich auch an anderen Stellen manifestieren, wie z. B. an retinalen Gefäßen [5].

Retinale Gefäßverschlüsse sind eine häufige Ursache für Visusminderungen. Man unterscheidet dabei arterielle und venöse Verschlüsse, es gibt jedoch auch Mischbilder. Ein retinaler Venenverschluss bzw. eine Venenthrombose ist als multifaktorielles Geschehen zu sehen und hängt stark mit altersbedingten lokalen und systemischen Faktoren zusammen. Die Ursachenabklärung und Therapie sind demnach in interdisziplinärer Zusammenarbeit zu sehen. Es wird angenommen, dass die Pathogenese des Venenverschlusses den Prinzipien der Virchow-Trias für die Thrombogenese folgt, die Gefäßschäden, Stase und Hyperkoagulabilität umfasst. Hierbei stellt im Allgemeinen die arteriosklerotische Verdickung eines Arteriolenasts an einer arteriovenösen Kreuzung eine wichtige Komponente bei der Entstehung des Venenverschlusses durch Kompression, die durch eine gemeinsame Adventitia verstärkt wird. Im Bereich der Lamina cribrosa teilen sich die zentrale Netzhautvene und die Arterie an den arteriovenösen Kreuzungen eine gemeinsame Adventitia, sodass hier durch atherosklerotische Veränderungen der Arterie ein Zentralvenenverschluss ausgelöst werden kann [3, 4]. Ein retinaler Arterienverschluss wird v. a. durch arteriosklerotische Embolien bzw. Thrombosen, Vaskulitiden oder Vasospasmus verursacht. Als Risikofaktoren für Gefäßverschlüsse gelten allgemein ein erhöhtes Lebensalter über 55 Jahre, arterielle Hypertonie, Hyperlipidämie, Diabetes mellitus, die Einnahme von oraler Kontrazeption, erhöhter Augeninnendruck sowie Erkrankungen des blutbildenden Systems oder bei entzündlichen Veränderungen, die eine Erhöhung der Blutviskosität bzw. Hyperkoagulabilität bedingen. Thrombophilien sind v. a. bei jüngeren Patienten von Bedeutung.

In diesem Fallbericht liegt ein Mischbild aus arteriovenösem Gefäßverschluss bei einem ansonsten gesunden 77-jährigen Patienten vor. Wie bereits dargelegt, wird von einem immunologischen Ereignis insbesondere einer autoimmunbedingten, heparininduzierten Thrombozytopenie als Ursache für die Thromboseneigung im Rahmen der Impfung ausgegangen. Eine Thrombozytopenie lag jedoch bei dem hier dargestellten Patienten nicht vor, auch traten keine anderen thrombotischen Ereignisse wie die häufig berichteten Hirnvenenthrombosen nach Impfung auf, und das Alter von 77 Jahren liegt weitaus höher als in der betroffenen Patientenpopulation. Es ist insbesondere im Hinblick auf die Pathogenese eines venösen Gefäßverschlusses fraglich, ob die Impfung von Vaxzevria® (AstraZeneca) tatsächlich ursächlich für den Gefäßverschluss des Patienten war.

## Fazit für die Praxis

Ursachen für retinale Gefäßverschlüsse, v. a. im venösen Gefäßsystem, werden trotz genauer internistischer Abklärung häufig nicht gefunden, dies kann v. a. für den Patienten sehr frustrierend sein. Ob der Pathomechanismus, der durch die Impfung von Vaxzevria® (AstraZeneca) ausgelöst werden kann, auch einen retinalen arteriovenösen Gefäßverschluss bedingen kann oder es sich hier um einen Zufall handelt, ist nach aktueller Datenlage eher unwahrscheinlich. Vorsicht ist vor dem voreiligen Ziehen von Zusammenhängen geboten, da die beschriebenen thromboembolischen Ereignisse nach Impfung weiterhin sehr selten sind, in der Regel bei 20- bis 63-Jährigen etwa 4 bis 16 Tage später auftreten und im Gegensatz zu unserem 77-jährigen Patienten stehen, bei dem die Symptome bereits nach 1 Tag nach Impfung auftraten. Außerdem ist von einem Vorliegen kardiovaskulärer Risikofaktoren bei unserem Patienten auszugehen aufgrund fundoskopisch gesicherter Kreuzungszeichen und Tortuositas der retinalen Gefäße.

Zusammenfassend kann eher von einem Zufall des zeitlichen Zusammenhangs zwischen Impfung und Auftreten des Gefäßverschlusses ausgegangen werden. Vor allem im Hinblick auf die derzeitigen steigenden Inzidenzzahlen sollte kein Zweifel am Risiko-Nutzen-Verhältnis der Impfstoffe aufkommen.
